# A Prospective Assessment of the Accuracy of Commercial IgM ELISAs in Diagnosis of Japanese Encephalitis Virus Infections in Patients with Suspected Central Nervous System Infections in Laos

**DOI:** 10.4269/ajtmh.2012.11-0729

**Published:** 2012-07-01

**Authors:** Catrin E. Moore, Stuart D. Blacksell, Thaksinaporn Taojaikong, Richard G. Jarman, Robert V. Gibbons, Sue J. Lee, Vilada Chansamouth, Soulignasack Thongpaseuth, Mayfong Mayxay, Paul N. Newton

**Affiliations:** Wellcome Trust-Mahosot Hospital-Oxford Tropical Medicine Research Collaboration, Mahosot Hospital, Vientiane, Lao PDR; Centre for Clinical Vaccinology and Tropical Medicine, Nuffield Department of Clinical Medicine, University of Oxford, Churchill Hospital, Oxford, United Kingdom; Faculty of Tropical Medicine, Mahidol University, Bangkok, Thailand; Armed Forces Research Institute of Medical Sciences, Bangkok, Thailand

## Abstract

Japanese encephalitis virus (JEV) is a major cause of encephalitis in Asia. We estimated the diagnostic accuracy of two anti-JEV immunoglobulin M (IgM) enzyme-linked immunosorbent assays (ELISAs) (Panbio and XCyton *JEV*CheX) compared with a reference standard (AFRIMS JEV MAC ELISA) in a prospective study of the causes of central nervous system infections in Laos. Cerebrospinal fluid (CSF; 515 patients) and serum samples (182 patients) from those admitted to Mahosot Hospital, Vientiane, were tested. The CSF from 14.5% of acute encephalitis syndrome (AES) patients and 10.1% from those with AES and meningitis were positive for anti-JEV IgM in the reference ELISA. The sensitivities for CSF were 65.4% (95% confidence interval [CI] = 51–78) (Xcyton), 69.2% (95% CI = 55–81) (Panbio), however 96.2% (95% CI = 87–100) with Panbio Ravi criteria. Specificities were 89–100%. For admission sera from AES patients, sensitivities and specificities of the Panbio ELISA were 85.7% (95% CI = 42–100%) and 92.9% (95% CI = 83–98%), respectively.

## Introduction

The Japanese encephalitis virus (JEV) is an important cause of encephalitis in Asia, with an estimated 35,000–50,000 cases and 10–15,000 deaths annually.^1^–^6^ Despite being surrounded by countries with documented JEV infections, there is very little information about JEV in the Lao People's Democratic Republic (Laos). Anti-JEV immunoglobulin M (IgM) has been described in the cerebrospinal fluid (CSF) of 5 of 26 patients with viral encephalitis in Vientiane hospitals (Innis and others, unpublished data) and anti-JEV antibodies occur in 50% of healthy adults in central Laos.^7^ There is no routine JEV vaccination in Laos, and there are insufficient data to inform Lao public health and vaccination policy.^8^–^12^

Diagnosis of Japanese encephalitis is difficult because it is clinically indistinguishable from other causes of acute encephalitis^8^,^13^ and there is serological cross-reaction with dengue virus and other flavivirus antibodies.^14^,^15^ Polymerase chain reaction (PCR) assays and cell culture are technically sophisticated and expensive, and although they are specific, have a low sensitivity as JEV is usually not detectable in admission blood and CSF.^13^ A dengue/JEV IgM antibody capture enzyme-linked immunosorbent assay (MAC-ELISA), developed by the United States Army Medical Component-Armed Forces Research Institute of Medical Sciences (AFRIMS),^16^–^18^ has become a reference serological assay in the region. However, this is not commercially available and there are no simple, accessible, and accurate tests to diagnose JEV infection in Laos.

Recently, commercial ELISAs for the detection of anti-JEV IgM antibodies have been developed.^15^ At least two such kits are currently available in Asia—the Panbio JEV-IgM Dengue Combo ELISA (Inverness Medical Innovations, Brisbane, Australia) and the XCyton *JEV* CheX (XCyton Diagnostics Ltd., Bangalore, India). The manufacturers claim that these ELISAs detect anti-JEV IgM with good sensitivity and specificity and that the Panbio JEV-IgM Dengue Combo ELISA is able to distinguish acute dengue and JEV infection. The detection of anti-JEV IgM in CSF is thought to be more specific for the diagnosis of acute JEV encephalitis than detection in sera because cross-reactive flavivirus antibodies are probably less likely to be found in CSF than sera and anti-JEV antibodies may persist longer in serum than in CSF.^19^ Serum-based IgM assays may also detect IgM antibodies resulting from JEV infections without encephalitis and persistence after JEV infection or vaccination. Commercial ELISA kits have been previously evaluated using a selected case series of sera and CSF.^1^,^14^,^15^ However, there is only one published investigation of the accuracy of commercial ELISAs in a prospective study with the clinical description of patients.^20^

There is an urgent need for data on the incidence of JEV encephalitis in Laos. We therefore prospectively examined the diagnostic accuracy of Panbio JEV Dengue IgM Combo ELISA and XCyton *JEV* CheX ELISA, in comparison with the reference AFRIMS JEV MAC ELISA, for the detection of anti-JEV IgM antibodies in CSF and serum in patients with suspected central nervous system (CNS) infections in Laos, where dengue and JEV co-circulate.

## Materials And Methods

### Patients.

Samples were collected at Mahosot Hospital, a 365 bed primary-tertiary hospital in Vientiane, Laos, between January 2001 and April 2008. The CSF was collected by lumbar puncture (LP) from patients with suspected CNS infection, according to the judgment of the responsible physician if the patient (or parent or guardian) gave written informed consent. We did not use a formal definition of CNS infection. Patients who the responsible physician felt may have a CNS infection and had no contraindications to LP were included. This series therefore included patients with acute encephalitis syndrome (AES) and meningitis. The AES was defined “as a person of any age, at any time of year with the acute onset of fever and either a change in mental status (including symptoms such as confusion, disorientation, coma, or inability to talk) and/or new onset of seizures (excluding simple febrile seizures).”^21^ Meningitis^22^ is defined by the World Health Organization (WHO) as a patient “with sudden onset of fever (> 38.5°C rectal or 38.0°C axillary) and one of the following signs: neck stiffness, altered consciousness, or other meningeal signs.”^21^ We adapted this definition, replacing “with sudden onset of fever (> 38.5°C rectal or 38.0°C axillary)” with “fever” as we saw patients, especially young children with clinical meningitis, but with temperatures below the WHO^21^ temperature criterion. The LPs were not routinely repeated.

Ethical clearance was granted by the Ethical Review Committee of the Faculty of Medical Sciences, National University of Lao, Vientiane, Laos and by the Oxford University Tropical Ethics Research Committee, Oxford, UK. The patient's history and clinical examination were recorded on a standard form. The CSF was immediately sent for cell count and conventional investigations^23^ and admission and convalescent (paired) sera collected. Aliquots of CSF and serum were immediately stored at −80°C.

### ELISAs.

#### Commercial ELISAs.

The two commercial ELISAs for the diagnosis of JEV were performed in Vientiane following the manufacturer's instructions, with plates read at 450 nm using a Multiskan EX ELISA plate reader (Labsystems, Franklin, MA). All plates were repeated if the positive, negative, or calibrator samples were out of range. All equivocal results were repeated. If the repeat result was also equivocal the sample was reported as negative. The CSF and serum aliquots were sent to the Department of Virology, AFRIMS, Bangkok for reference ELISA testing, without personal identifiers and blinded to the results obtained in Vientiane.

The Panbio Japanese Encephalitis Dengue IgM Combo ELISA (Cat. no. E-JED01C; Inverness Medical Innovations, Brisbane, Australia [formerly Panbio Ltd.]) detects anti-JEV and anti-dengue IgM antibodies in serum. This kit does not contain a CSF testing protocol and after consultation with Panbio Ltd. a working CSF dilution of 1:10 was used, the same as that applied by Ravi.^24^ Panbio units were calculated by multiplying the index value (calculated by dividing the sample absorbance by the cut-off value [the average absorbance of the three calibrators]) by 10. The results for dengue IgM and JEV IgM were expressed in “Panbio Units” as calculated from the sample absorbance (as explained in the kit instructions). We used two cutoffs to interpret the results. First, using the method described by Panbio in their instruction leaflet, the results were classed as negative for dengue and JEV if PanBio units were < 9, equivocal if 9–11 and positive if > 11. If both the dengue and JEV IgM results were positive, the JEV result was divided by the dengue result to give a ratio, with ≥ 1 indicating JEV infection and < 1 indicating dengue infection. Second, we used the cutoffs described by Ravi^24^ with Panbio units < 2 JEV negative, 2–4 equivocal and > 4 JEV positive, and for dengue < 12 dengue negative, 12–14 equivocal and > 14 positive for dengue. Using the Ravi criteria (henceforth referred to as “Panbio RC” as different from the standard interpretation “Panbio SI”), if both the dengue and JEV interpretations were positive the sample was classified as presumptive dengue infection.^24^

According to the manufacturer's instructions, the XCyton ELISA (XCyton Diagnostics Ltd., Bangalore, India) specifically detects anti-JEV, but not anti-dengue, IgM antibodies in serum, and CSF (at a 1:10 dilution). We only tested CSF and not serum samples using this kit. Samples with XCyton units < 30 units were classed as negative and ≥ 100 units were classed as JEV. The manufacturer's instructions state that 30–99 units are classed as “suspected recent flavivirus infection,” and samples with such results were classified as negative for the purposes of the JEV diagnostic performance evaluation.

#### AFRIMS in-house JEV/dengue MAC ELISA.

The 96-well microtiter plates (Linbro/Titertek E.I.A., MP Biomedicals Inc., Horsham, PA) were coated with rabbit anti-human IgM antibody (IgM and IgG, Kierkegaard & Perry Laboratory [KPL], Gaithersburg, MD). Fifty microliters of serum diluted at 1:100 in phosphate buffered saline (PBS) or CSF diluted at 1:10 in PBS was added to the wells and incubated overnight. The plates were washed and JEV antigen or tetravalent dengue antigen added to the plate.^17^ The plates were incubated and then washed and optimal diluted horseradish peroxidase (HRP)-conjugated anti-flavivirus IgG was added.^25^ The plates were incubated and o-phenylenediamine substrate was added for color development. The reaction was stopped by adding sulphuric acid and the absorbance was read at 490 nm using an ELISA reader. Patients with anti-dengue IgM units < 40 U and anti-JEV IgM units > 40 U were classified as having acute JEV infection. If a patient was positive for dengue and JEV, the ratio of anti-dengue/anti-JEV IgM were used with > 1 interpreted as dengue and < 1 as JEV.

### Statistical analysis.

The AFRIMS JE MAC ELISA result was used as the reference comparator. STATA v10 (College Station, TX) software was used to calculate sensitivity, specificity, positive predictive values (PPV), and negative predictive values (NPV), with 95% confidence intervals (CIs), and receiver operating characteristic curves (ROCC). The sensitivity and specificity for JEV alone were estimated, and then cross-reactions between dengue and JEV examined. The Standards for Reporting of Diagnostic Accuracy (STARD) reporting guidelines were used.^26^

### Results

#### Patients.

**Figure 1.**
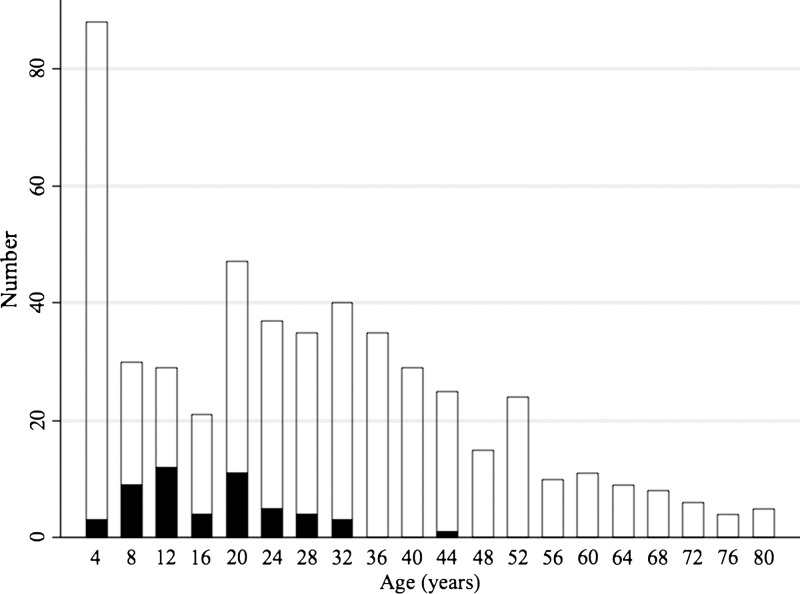
Age distribution for patients with suspected central nervous system (CNS) infection and cerebrospinal fluid (CSF) samples available (*N* = 515). The black bars represent CSF samples AFRIMS JE MAC ELISA positive and the clear bars represent CSF samples that were AFRIMS JE MAC ELISA negative. Patients were grouped into ages spanning 4 years (where 0–4 years are in the bar labeled 4, 5–8 years are in the bar labeled 8, 9–12 years are in the bar labeled 12 up to the bar labeled 80, which contains all adults over 80 years of age).

**Figure 2.**
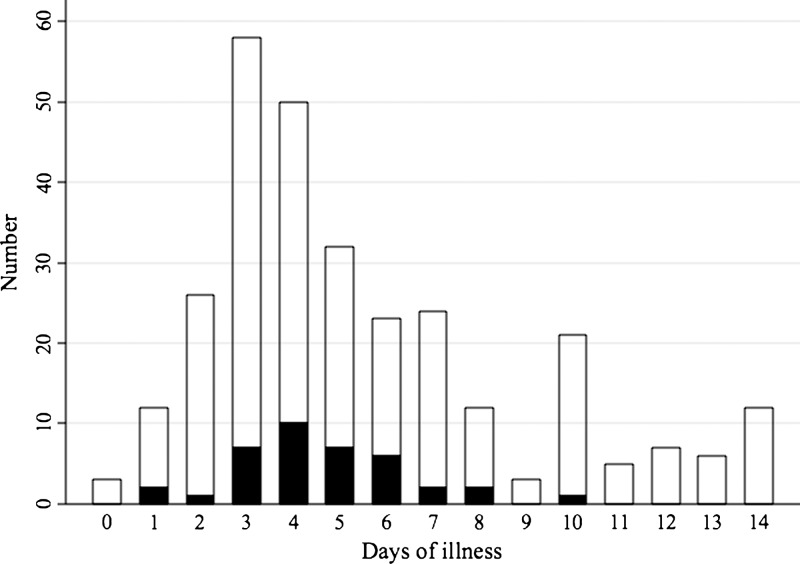
Number of days of illness at admission for all patients with illness recorded as < 15 days before admission (*N* = 299 of 515; 58%) and for those with anti-JEV IgM antibodies detected in cerebrospinal fluid (CSF) using the AFRIMS JE MAC ELISA (total is 38 of 299; 12.7%). The black bars represent CSF samples AFRIMS JE MAC ELISA positive and the clear bars represent CSF samples AFRIMS JE MAC ELISA negative.

Between January 2001 and April 2008, 578 patients with suspected CNS infection were admitted to Mahosot Hospital. Because lumbar puncture could not be performed for 36 (6%) patients and there was insufficient CSF available after culture for 27 (5%) patients, 515 (89%) patients were included. Using the WHO (2003)^21^ criteria, 234 (45.4%) patients had AES, whereas 256 (49.7%) had meningitis using the modified WHO (2003) definition (above) and 157 (30.5%) patients had both AES and meningitis. The median (interquartile range [IQR]; range) age of patients was 24 (8–38; 0.05–85) years and 32% were < 15 years of age (Figure 1, Table 1). Patients predominantly came from Vientiane City and Province (84%) and presented with a median of 4 days of fever (Figure 2). Headache, neck stiffness, convulsions, and reduced Glasgow Coma Score (GCS < 15) were present for 77%, 60%, 29%, and 46% of patients, respectively. The median (range) interval between paired sera was 8 (1–73) days. Of those with anti-JEV IgM detected in CSF by AFRIMS ELISA, 42% had convulsions before admission, 63% had a reduced GCS, the median (range) CSF white cell count was 125 (0–653)/μL with a median percentage of lymphocytes in CSF of 37 (0–90)%. Mortality in the hospital for all those with anti-JEV IgM detected in CSF was 4% (Table 1).

#### Anti-JEV and anti-dengue IgM ELISA results for CSF.

Of the 515 CSF samples examined using the AFRIMS ELISA, 52 of 515 (10.1%) patients had anti-JEV IgM antibodies detected, 34 (14.5%) with AES, 34 (13.3%) with meningitis, and 28 (17.8%) with both AES and meningitis. Of 95 (37.1%) patients who had meningitis, but AES was negative (4 patients had no record of AES status), 6 (6.3%) had anti-JEV IgM detected in CSF. The highest sensitivity for the detection of JEV IgM in CSF was yielded by the Panbio RC (96.2% and NPV of 99.5%) and the lowest was with the XCyton ELISA (65.4% and NPV of 96.3%) (Table 2). The highest specificity (99.8%) and PPV (97.1%) were yielded by the XCyton ELISA and the lowest with the Panbio RC (specificity 88.8% and PPV 49.0%) (Table 2). Of the 515 CSF samples examined, 299 had < 15 days of illness recorded before admission. The proportion of JEV-positive patients rose to a maximum of 15.3% (20 of 131) at Day 4 and then declined to zero at Day 11 (Figure 2).

For the 234 patients with AES, when compared with the AFRIMS ELISA, the commercial ELISAs showed sensitivities for detection of anti-JEV IgM in CSF between 76.5% and 97.1%, with the highest being for Panbio RC. Specificities were between 89% and 100%, with the highest for the XCyton ELISA (Table 2); the lowest PPV was observed for the Panbio RC (60%) and the highest with the XCyton ELISA (100%). For the 256 patients with meningitis, a similar pattern of results was noted; the test ELISAs showed sensitivities ranging between 67.6% and 97.1%, specificities between 84.7% and 99.5% (Table 2); the lowest PPV was observed with the Panbio RC (49%) and the highest with the XCyton ELISA (96%).

The ROCCs were calculated to determine the optimal diagnostic cutoffs for each test using CSF (Table 3) when compared with the AFRIMS JE MAC ELISA. For the Panbio ELISA the optimal cutoff was 4.4 Panbio units determined from the best compromise of sensitivity (92.3%) and specificity (91.1%) and for the XCyton ELISA the optimal cutoff was 28.8 with a sensitivity (94.2%) and specificity (99.1%).

Anti-dengue IgM was detected in CSF from five patients (5 of 515; 1.0%) by the AFRIMS MAC ELISA. Four patients were classified as having meningitis and none as AES. One patient with anti-dengue IgM antibodies by the AFRIMS MAC ELISA was also positive for anti-JEV IgM antibodies in the Panbio ELISA (16.0 Panbio units for JEV IgM and 3.7 Panbio units for dengue IgM) using both the PanBio SI and modified Panbio RC. An additional two patients with anti-dengue IgM antibodies detected by AFRIMS MAC ELISA were positive for anti-JEV IgM antibodies by the Panbio RC ([patient 1] 6.7 JEV Panbio units together with 3.3 Dengue Panbio units, and [patient 2] 7.4 JEV Panbio units together with 8.5 Dengue Panbio units).

#### Dengue and JEV ELISAs using serum.

Of the 182 (182 of 515; 35.3%) CNS patients with serum samples examined, acute serum samples were collected from 166 patients, with convalescent serum samples from 129 patients and paired sera from 113 patients. Of 182 patients with sera available, 74 (40.7%) had AES and 76 (41.8%) had meningitis (Supplemental Table). For all sera, the sensitivity and specificity of the Panbio SI ELISA, in comparison to the AFRIMS JE MAC ELISA, were 70.8% (95% CI = 49–87%) and 94.9 (95% CI = 90–98%). For patients with AES, sensitivity was 87.5% (95% CI = 62–98%) and specificity was 93.1% (95% CI = 83–98%) (Supplemental Table). For admission sera from AES patients, sensitivities and specificities of the Panbio ELISA were 85.7% (95% CI = 42–100%) and 92.9% (95% CI = 83–98%), respectively.

Of the 113 patients with paired specimens, using the AFRIMS JE MAC ELISA 16 (14.2%) patients were positive for anti-JEV IgM, including 10 (62.5%) with AES, 10 (62.5%) with meningitis and 8 (50.0%) patients with both AES and meningitis. Using the AFRIMS JE MAC ELISA 7 (6.2%) patients seroconverted to anti-JEV IgM positivity, whereas using the Panbio SI five of these patients seroconverted and three did not.

ROCCs were calculated for the Panbio ELISA using serum and the optimal diagnostic cutoff was 4.97 Panbio units (sensitivity 85.7%; specificity 75.8%) when compared with the AFRIMS JE MAC ELISA (Table 3).

Of the 182 patients with admission and/or convalescent sera, 3 (1.6%) patients had anti-dengue IgM detected in either sample by the AFRIMS ELISA and the Panbio ELISA. A further 15 patients were negative for dengue in the AFRIMS ELISA (two were JEV IgM positive in the AFRIMS JE MAC ELISA), but positive in the Panbio ELISA.

#### Combined sera and CSF ELISA results in the diagnosis of JEV.

Using both admission and convalescent sera and the CSF results combined, 30 patients (30 of 182; 16.4%) were positive using the AFRIMS JE MAC ELISA. In the AFRIMS JE MAC IgM ELISA, of 74 patients with AES 19 (25.7%) were positive and of 76 with meningitis 18 (23.7%) were positive. In comparison, in the Panbio SI ELISA, the sensitivity and specificity were 76.7% (95% CI = 58–90%) and 96.7% (95% CI = 93–99%), respectively. For patients with AES, sensitivity was 89.5% (95% CI = 67–99%) and specificity was 96.4% (95% CI = 88–100%) (Supplemental Table). The PPV and NPV for AES patients were 89.5% (95% CI = 67–99%) and 96.4% (95% CI = 88–100%), respectively (Supplemental Table).

Combining the results of CSF with admission sera only, 23 (23 of 166; 13.9%) were positive using the AFRIMS JE MAC ELISA. Of 63 patients with AES, 13 (20.6%) had anti-JEV IgM and of 66 with meningitis, 13 (19.7%) had JEV IgM detected. Using the Panbio SI ELISA the sensitivity and specificity were 69.6% (95% CI = 47–87%) and 97.2% (95% CI = 93–99%), respectively (AES: sensitivity 84.6%; 95% CI = 55–98%: specificity 96.0%; 95% CI = 86–100%) (Supplemental Table). The PPV and NPV for AES were 84.6% (95% CI = 55–98%) and 96.0% (95% CI = 86–100%), respectively (Supplemental Table). Similar results were obtained by combining CSF with convalescent sera (taken median [range] of 8 [1–73] days after admission sera) (Supplemental Table).

#### Comparison of CSF and sera for the diagnosis of JEV in AES patients.

Of the 113 patients with paired sera and CSF, for patients with AES (*N* = 35), nine (25.7%) were positive in both CSF and sera. None and one (2.9%) patients were diagnosed by CSF alone and sera alone, respectively, using the AFRIMS JE MAC ELISA. Using only admission sera and CSF in the AFRIMS JE MAC ELISA, for those with AES (*N* = 63) seven (11.1%) patients were positive in both CSF and admission sera, a further six patients were positive in CSF alone (6 of 63 = 9.5%) and no patients positive in sera alone.

Using the Panbio SI, for 35 AES patients with paired sera and CSF, five (14.3%) patients were positive in all samples with a further two (5.7%) positive in CSF alone and two (5.7%) positive in sera alone. Using only admission sera and CSF for 131 patients with AES, 21 (16.0%) patients were positive in both sample types, six (4.6%) in CSF alone, and five (3.8%) in sera alone.

### Discussion

This is the first description of the clinical epidemiology of JEV infection in patients with CNS disease and the first evaluation of the performance of commercial ELISAs for the diagnosis of JEV infection in Laos.

Both the Panbio (Panbio SI) and XCyton ELISAs, when used to detect anti-JEV IgM in CSF alone, gave moderate to good sensitivity and excellent specificity when compared with the AFRIMS JE MAC ELISA, which is the reference gold standard in the region and has been validated for CSF and serum samples.^16^,^17^ However, when the “Ravi criterion” was applied to the Panbio assay (Panbio RC), sensitivities dramatically increased albeit with a reduction in specificity, for both AES and meningitis patients.

The ROCC analysis gave cutoff results similar to those recommended for CSF (XCyton and the Panbio RC) and these diagnostic cutoffs appear appropriate for Laos. However, the ROCC analysis suggested that the optimal diagnostic cutoff for serum in Laos should be 4.97 Panbio units, rather than 4.0. Although this cutoff gave high accuracy in this Laos series, further studies are required in other settings to determine its regional applicability. This study suggests that acute and convalescent sera are comparable to CSF in terms of classifying AES patients as having JEV using the reference ELISA. However, if admission sera alone had been used, 9.5% of AES patients would not have been diagnosed with JEV (the CSF was positive and the admission serum was negative). Sera are easier to collect than CSF samples as skilled invasive LP insertion is not required. However, convalescent sera are difficult to collect in the rural tropics because of the difficulties of patient follow-up.

Limitations of this study include that we did not evaluate the XCyton ELISA with sera and that the surveillance was hospital-based in the capital city and will therefore not accurately reflect the situation in more distant rural Laos. Lumbar puncture and CSF analysis is available in Vientiane, but not elsewhere in the country. The population from which these JEV-positive patients arose is unclear and a minority of patients had sera available, as a result of it being used for other tests and the difficulties of follow-up. The JEV vaccination became available in Vientiane during this study, however on a very small scale, and it is extremely unlikely that this confounded the diagnoses. In Vietnam, one-third of JEV-infected children presented with acute limb paralysis, meningitis, or both, and therefore would not necessarily have been detected by the current AES case definition.^13^ Consequently, although surveillance and investigation of AES is important in understanding JEV epidemiology, it will underestimate the burden of severe JEV associated disease.

The results presented here are similar to those from retrospective studies of selected case series and the one prospective evaluation (Table 4). Three different in-house assays have been used as reference tests in these evaluations but their diagnostic accuracies have not been compared. One study examined the XCyton and PanBio kits with CSF from Indian patients with AES, against the Venture Technologies ELISA, and described sensitivities of 68–75% and specificities of 97%.^20^ However, a comparison of PanBio and XCyton kits, versus CDC assays, in samples from Indian and Bangladeshi patients with AES and meningitis, found a high specificity, however a much lower sensitivity (20–60%) than that described here and in previous reports.^20^,^27^ The reasons for this are not clear.

These data suggest that the Panbio and XCyton ELISAs are accurate tools for the diagnosis of JEV in patients with suspected CNS infections in Laos. Although accessible and subject to quality control they are relatively expensive (~350–430 US$ per kit or 4–10 US$/sample assay) and require trained technicians and relatively expensive ELISA readers. The JEV is an important preventable cause of CNS infections in Laos and the expanded use of ELISA assays nationally would help define the burden of disease. These data suggest that JEV vaccination should be considered.

## Supplementary Material

Supplemental Table

## Figures and Tables

**Table 1:** Demographic and clinical features of 515 patients with suspected central nervous system infection with cerebrospinal fluid (CSF) examined

	All patients	AES	Meningitis	Anti-JEV IgM detected in CSF by AFRIMS JE MAC ELISA
Female (%)	186^515^ (36)	93^234^ (40)	86^256^ (34)	19^52^ (37)
Age	24^515^ (8–38; 0.05–85)	17^234^ (3–35; 0.1–85)	24.5^256^ (14–40; 0.25–80)	12.5^52^ (8–12.5; 2–40)
Aged < 15 years (%)	166^515^ (32)	109^234^ (47)	68^256^ (27)	28^52^ (54)
Aged < 5 years (%)	97^515^ (19)	69^234^ (30)	18^256^ (7)	8^52^ (15)
Home in Vientiane City (%)	350^511^ (69)	151^233^ (65)	170^253^ (67)	23^52^ (44)
Home in Vientiane Province (%)	81^511^ (16)	41^233^ (18)	44^253^ (17)	11^52^ (21)
Days of fever	4^430^ (2–10)	4^200^ (3–7)	5^209^ (3–10)	6.7^45^ (4–6)
Vomiting (%)	231^483^ (48)	115^225^ (51)	145^240^ (60)	30^49^ (61)
Headache (%)	370^483^ (77)	158^225^ (70)	240^240^ (100)	43^49^ (88)
Neck stiffness (%)	291^484^ (60)	171^225^ (76)	212^240^ (88)	36^49^ (74)
Confusion (%)	165^483^ (34)	109^224^ (49)	103^239^ (43)	22^48^ (46)
Convulsions (%)	139^483^ (29)	117^224^ (52)	57^239^ (24)	20^48^ (42)
Admission body temperature* °C	38.4^467^ (38.5–38.5)	38.7^215^ (38.6–38.8)	38.9^225^ (38.8–39.0)	38.7^49^ (38.4–38.9)
GCS/15	12.7^449^ (10–15)	10.5^208^ (8–13)	12.1^227^ (10–15)	11.9^48^ (10–15)
Number with GCS < 15 (%)	205^449^ (46)	179^208^ (86)	132^227^ (58)	30^48^ (63)
CSF pressure mm H_2_0	20^479^ (15–30; 3–100)	20^220^ (14–27; 3–100)	20^240^ (15.8–30.8; 3–100)	22^50^ (15–25.5; 5–100)
CSF white cell count/μL	168^492^ (8–130; 0–9600)	212^226^ (10–155; 0–9600)	240^245^ (15–285; 0–9600)	125^51^ (25–155; 0–653)
CSF neutrophils (%)	50^484^ (20–80; 0–100)	56^219^ (25–89; 0–100)	50^245^ (25–80; 0–100)	57^50^ (27–89; 0–100)
CSF lymphocytes (%)	37^481^ (2–60; 0–100)	36^217^ (4–59; 0–100)	40^244^ (11–66; 0–100)	37^50^ (7–67; 0–90)
Number with anti-JEV IgM in serum (%)	24^183^ (13)	16^85^ (19)	15^80^ (19)	20^27^ (74)
Number with anti-JEV IgM in CSF (%)	52^515^ (10)	34^234^ (15)	34^256^ (13)	–
Died in hospital (%)	48^413^ (12)	35^200^ (18)	23^216^ (11)	2^45^ (4)

Median (interquartile range [IQR]; range), except admission body temperature*, which is mean (95% confidence interval). For continuous variables sample size is given as superscript to take into account missing values. AES and meningitis as defined in Methods.

GCS = Glasgow Coma Score. (%) is the percentage of samples that are positive for each observation.

**Table 2:** Sensitivity and specificity in detection of anti-JEV IgM of two commercial ELISAs compared with the AFRIMS JE MAC ELISA for cerebrospinal fluid (CSF) samples

Test ELISA kits on CSF samples		AFRIMS JE MAC ELISA results for CSF (reference)		Diagnostic accuracy (95% CI)			
		Positive (%)	Negative (%)	% Sensitivity	% Specificity	PPV	NPV
All patients (*N* = 515)		52 (10.1)	463 (89.9)				
XCyton	Positive	34 (6.6)	1 (0.2)	65.4 (51–78)	99.8 (99–100)	97.1 (85–100)	96.3 (94–98)
	Negative	18 (3.5)	462 (89.7)
Panbio SI	Positive	36 (7.0)	5 (1.0)	69.2 (55–81)	98.9 (98–100)	87.8 (74–96)	96.6 (95–98)
	Negative	16 (3.1)	458 (88.9)
Panbio RC†	Positive	50 (9.7)	52 (10.1)	96.2 (87–100)	88.8 (86–92)	49.0 (39–59)	99.5 (98–100)
	Negative	2 (0.4)	411 (79.8)
Patients with AES (*N* = 234)		34 (14.5%)	200 (85.5%)				
XCyton	Positive	26 (11.1)	0	76.5 (59–89)	100 (98–100)	100 (87–100)	96.2 (93–98)
	Negative	8 (3.4)	200 (85.5)
Panbio SI	Positive	29 (12.4)	2 (0.9)	85.3 (69–95)	99.0 (96–100)	93.5 (79–99)	97.5 (94–99)
	Negative	5 (2.1)	198 (84.6)
Panbio RC†	Positive	33 (14.1)	22 (9.4)	97.1 (85–100)	89.0 (84–93)	60.0 (46–73)	99.4 (97–100)
	Negative	1 (0.4)	178 (76.1)
Patients with meningitis (*N* = 256)		34 (13.3%)	222 (86.7%)				
XCyton	Positive	23 (9.0)	1 (0.4)	67.6 (50–83)	99.5 (98–100)	95.8 (79–100)	95.3 (92–98)
	Negative	11 (4.3)	221 (86.3)
Panbio SI	Positive	26 (10.2)	4 (1.6)	76.5 (59–89)	98.2 (96–100)	86.7 (69–96)	96.5 (93–99)
	Negative	8 (3.1)	218 (85.2)
Panbio RC†	Positive	33 (12.9)	34 (13.3)	97.1 (85–100)	84.7 (79–89)	49.3 (37–62)	99.5 (97–100)
	Negative	1 (0.4)	188 (73.4)

* CI = confidence interval, *N* is the total number of CSF samples tested for each group.

† Revised cutoff for CSF.^24^

**Table 3:** Area under receiver operator characteristic curves (AUROCCs) for optimal diagnostic cutoffs using samples from Lao central nervous system (CNS) disease patients*

Samples	ELISA	ROCC cutoffs	Sensitivity % (95% CI)	Specificity % (95% CI)	AUROCC 95% CI
i) CSF (*N* = 515)					
	Panbio	4.44	92.3 (82–98)	91.1 (88–94)	0.97 (0.95–0.99)
	XCyton	28.76	94.2 (84–99)	99.1 (98–100)	0.99 (0.97–1.00)
ii) Sera (*N* = 182)					
	Panbio	4.97	85.7 (63–97)	75.8 (68.4–82.2)	0.83 (0.74–0.93)

* i) For 515 cerebrospinal fluid (CSF) samples tested in the Panbio (Panbio Units) and the XCyton ELISA compared with the reference AFRIMS JEV ELISA, and ii) for 182 serum samples tested in the Panbio ELISA (Panbio Units) results compared with AFRIMS JEV ELISA.

ELISA = enzyme-linked immunosorbent assay; ROCC = receiver operating characteristic curves; CI = confidence interval.

**Table 4:** Summary of comparisons of commercial ELISAs for detection of anti-JEV IgM in CSF and sera with reference assays

Study type	Sample type (number)	Kit name	Reference ELISA											
			CSF						Serum					
			JEV pos	JEV neg	% Sensitivity (95% CI)	% Specificity (95% CI)	PPV (%)	NPV (%)	JEV pos	JEV neg	% Sensitivity (95% CI)	% Specificity (95% CI)	PPV (%)	NPV (%)
Ravi^24^Retrospective, India*	CSF (60)	XCyton	18/2	1/39	90 (70–97)	97.5 (87–100)	95	95	–	–	–	–	–	–
		Panbio	13/7	2/38	65 (43–82)	95 (84–99)	87	84	–	–	–	–	–	–
		Panbio RC	16/4	2/38	80 (58–92)	95 (84–99)	89	91	–	–	–	–	–	–
Jacobson^15^ Retrospective, Nepal and Thailand†	Acute serum (360)	XCyton	–	–	–	–	–	–	117/4	83/156	96.7 (92–99)	65.3 (59–71)	59	98
		Panbio	–	–	–	–	–	–	108/13	2/237	89.3 (82–94)	99.2 (97–100)	98	95
		InBios	–	–	–	–	–	–	120/1	105/134	99.2 (96–100)	56.1 (50–63)	53	99
Lewthwaite^20^ Prospective, India‡	CSF (105) Serum (159)	XCyton	21/6	2/75	75.0 (57–87)	97.4 (91–99)	91	91	25/15	1/118	57.3 (46–68)	98.4 (96–100)	93	85
		Panbio	18/9	2/73	67.9 (49–82)	97.4 (91–99)	90	89	29/11	3/116	69.3 (58–79)	97.4 (94–99)	91	89
Khalakdina^1^ Retrospective, Nepal†	Serum (350)	XCyton	–	–	–	–	–	–	123/23	9/195	93 (88–97)	89 (85.0–93.0)	84	96
		Panbio1	–	–	–	–	–	–	94/11	38/207	71.0 (63–79)	95 (91–98)	90	85
		Panbio2	–	–	–	–	–	–	106/7	26/211	80.0 (72–87)	97.0 (94–99)	94	89
Robinson^27^ Prospective, Bangladesh and India*	CSF (226) Serum (294)	XCyton	6/24	1/195	20 (8–39)	99.5 (97–100)	86	89	24/37	4/229	39.3 (27–53)	98.3 (96–9100)	86	86
	CSF (184) Serum (270)	Panbio	9/8	2/165	52.9 (28–77)	98.8 (96–100)	82	95	29/22	10/209	56.9 (42–71)	95.4 (92–98)	74	91
	CSF (226) Serum (294)	InBios	5/25	5/191	16.7 (6–35)	97.4 (94–99)	50	88	12/49	7/226	19.7 (11–32)	97 (94–99)	63	82

Reference ELISAs used: *CDC = Centers for Disease Control and Prevention; †AFRIMS JE MAC ELISA; ‡VT reference Venture Technologies ELISA; A single study used the same reference standard as the current study.^15^

MAC-ELISA = IgM antibody capture enzyme-linked immunosorbent assay; CI = confidence interval; CSF = cerebrospinal fluid; CNS = central nervous system; PPV = positive predictive value; NPV = negative predictive value, pos/neg are the number positive over the number negative.

## References

[1] Khalakdina A, Shrestha SK, Malla S, Hills S, Thaisomboonsuk B, Shrestha B, Gibbons RV, Jacobson J (2010). Field evaluation of commercial Immunoglobulin M antibody capture ELISA diagnostic tests for the detection of Japanese encephalitis virus infection among encephalitis patients in Nepal. Int J Infect Dis.

[2] Solomon T, Kneen R, Dung NM, Khanh VC, Thuy TT, Ha DQ, Day NP, Nisalak A, Vaughn DW, White NJ (1998). Poliomyelitis-like illness due to Japanese encephalitis virus. Lancet.

[3] Erlanger Te, Weiss S, Keiser J, Utzinger J, Wiedenmayer K (2009). Past, Present, And Future Of Japanese Encephalitis. Emerg Infect Dis.

[4] Edelman R, Schneider RJ, Chieowanich P, Pornpibul R, Voodhikul P (1975). The effect of dengue virus infection on the clinical sequelae of Japanese encephalitis: a one year follow-up study in Thailand. Southeast Asian J Trop Med Public Health.

[5] Ding D, Hong Z, Zhao SJ, Clemens JD, Zhou B, Wang B, Huang MS, Zeng J, Guo QH, Liu W, Tao FB, Xu ZY (2007). Long-term disability from acute childhood Japanese encephalitis in Shanghai, China. Am J Trop Med Hyg.

[6] Tsai T (2000). New initiatives for the control of Japanese encephalitis by vaccination: minutes of a WHO/CVI meeting, Bangkok, Thailand, 13–15 October 1998. Vaccine.

[7] Vongxay P, Makino Y, Kanemura K, Saito M, Fukunaga T (1995). Seroepidemiology study of arbovirus infections in Khammouane Province, Lao PDR. Ryuku Med J.

[8] Chhour YM, Ruble G, Hong R, Minn K, Kdan Y, Sok T, Nisalak A, Myint KSA, Vaughn DW, Endy TP (2002). Hospital-based diagnosis of hemorrhagic fever, encephalitis, and hepatitis in Cambodian children. Emerg Infect Dis.

[9] Ketel WB, Ognibene AJ (1971). Japanese B encephalitis in Vietnam. Am J Med Sci.

[10] Kumar R, Tripathi P, Singh S, Bannerji G (2006). Clinical features in children hospitalized during the 2005 epidemic of Japanese encephalitis in Uttar Pradesh, India. Clin Infect Dis.

[11] Lowry PW, Truong DH, Hinh LD, Ladinsky JL, Karabatsos N, Cropp CB, Martin D,  Gubler DJ (1998). Japanese encephalitis among hospitalized pediatric and adult patients with acute encephalitis syndrome in Hanoi, Vietnam 1995. Am J Trop Med Hyg.

[12] Srey VH, Sadones H, Ong S, Mam M, Yim C, Sor S, Grosjean P, Reynes JM (2002). Etiology of encephalitis syndrome among hospitalized children and adults in Takeo, Cambodia, 1999–2000. Am J Trop Med Hyg.

[13] Solomon T, Thao TT, Lewthwaite P, Ooi MH, Kneen R, Dung NM, White N (2008). A cohort study to assess the new WHO Japanese encephalitis surveillance standards. Bull World Health Organ.

[14] Ravi V, Desai A, Balaji M, Apte MP, Lakshman L, Subbakrishna DK, Sridharan G, Dhole TN, Ravikumar BV (2006). Development and evaluation of a rapid IgM capture ELISA (JEV-Chex) for the diagnosis of Japanese encephalitis. J Clin Virol.

[15] Jacobson JA, Hills SL, Winkler JL, Mammen M, Thaisomboonsuk B, Marfin AA, Gibbons RV (2007). Evaluation of three immunoglobulin M antibody capture enzyme-linked immunosorbent assays for diagnosis of Japanese encephalitis. Am J Trop Med Hyg.

[16] Burke D, Nisalak A (1982). Detection of Japanese encephalitis virus immunoglobulin M antibodies in serum by antibody capture radioimmunoassay. J Clin Microbiol.

[17] Burke D, Nisalak A, Ussery M (1982). Antibody capture immunoassay detection of Japanese encephalitis virus immunoglobulin M and G antibodies in cerebrospinal fluid. J Clin Microbiol.

[18] Innis BL, Nisalak A, Nimmannitya S, Kusalerdchariya S, Chongswasdi V, Suntayakorn S, Puttisri P, Hoke CH (1989). An enzyme-linked immunosorbent assay to characterize dengue infections where dengue and Japanese encephalitis co-circulate.. Am J Trop Med Hyg.

[19] Burke DS, Nisalak A, Ussery MA, Laorakpongse T, Chantavibul S (1985). Kinetics of IgM and IgG responses to Japanese encephalitis virus in human serum and cerebrospinal fluid. J Infect Dis.

[20] Lewthwaite P, Shankar M, Tio P, Daly J, Last A, Ravikumar R, Desai A, Ravi V, Cardosa J, Solomon T (2010). Evaluation of two commercially available ELISAs for the diagnosis of Japanese encephalitis applied to field samples. Trop Med Int Health.

[21] World Health Organization W (2003). Recommended standards for surveillance of selected vaccine-preventable diseases WHO/V&B/03.0. www.who.int/vaccines-documents/.

[22] Kuwayama M, Ito M, Takao S, Shimazu Y, Fukuda S, Miyazaki K, Kurane I, Takasaki T (2005). Japanese encephalitis virus in meningitis patients, Japan. Emerg Infect Dis.

[23] Moore CE, Sengduangphachanh A, Thaojaikong T, Sirisouk J, Foster D, Phetsouvanh R, McGee L, Crook DW, Newton PN, Peacock SJ (2010). Enhanced determination of *Streptococcus pneumoniae* serotypes associated with invasive disease in Laos by using a real-time polymerase chain reaction serotyping assay with cerebrospinal fluid. Am J Trop Med Hyg.

[24] Ravi V, Robinson JS, Russell BJ, Desai A, Ramamurty N, Featherstone D, Johnson BW (2009). Evaluation of IgM antibody capture enzyme-linked immunosorbent assay kits for detection of IgM against Japanese encephalitis virus in cerebrospinal fluid samples. Am J Trop Med Hyg.

[25] Bundo K, Igarashi A (1985). Antibody-capture ELISA for detection of immunoglobulin M antibodies in sera from Japanese encephalitis and dengue hemorrhagic fever patients. J Virol Methods.

[26] Bossuyt PM, Reitsma JB, Bruns DE, Gatsonis CA, Glasziou PP, Irwig LM, Lijmer JG, Moher D, Rennie D, de Vet HC (2003). Towards complete and accurate reporting of studies of diagnostic accuracy: the STARD initiative. BMJ.

[27] Robinson JS, Featherstone D, Vasanthapuram R, Biggerstaff BJ, Desai A, Ramamurty N, Chowdhury AH, Sandhu HS, Cavallaro KF, Johnson BW (2010). Evaluation of three commercially available Japanese encephalitis virus IgM enzyme-linked immunosorbent assays. Am J Trop Med Hyg.

