# Supplementary material for: A Prospective Assessment of the Accuracy of Commercial IgM ELISAs in Diagnosis of Japanese Encephalitis Virus Infections in Patients with Suspected Central Nervous System Infections in Laos
Source: Am J Trop Med Hyg. 2012 Jul 1;87(1):171–8. doi: 10.4269/ajtmh.2012.11-0729 (PMC3391045; doi:10.4269/ajtmh.2012.11-0729)
Supplement: Supplemental Table [file SD5.pdf]

Supplemental Table

AFRIMS JEV MAC ELISA results compared with Panbio ELISA results for detection of anti-JEV in CSF and serum (admission or convalescent serum)

Test results		AFRIMS JEV ELISA Positive (%)	AFRIMS JEV ELISA Negative (%)	CSF samples % Sens	CSF samples % Spec	CSF samples %PPV	CSF samples %NPV	Serum samples % Sens	Serum samples % Spec	Serum samples %PPV	Serum samples %NPV	CSF and Serum combined % Sens	CSF and Serum combined % Spec	CSF and Serum combined %PPV	CSF and Serum combined %NPV
CSF (patients with serum collections) (N = 182)		26 (14.2)													
Panbio SI criteria	Positive	19 (10.4)	0	73.1	100	100	95.7								
	Negative	7 (3.8)	156 (85.7)	(52–88)	(98–100)	(82–100)	(91–98)								
Panbio RC criteria	Positive	25 (13.7)	12 (6.6)	96.2	92.3	67.6	99.3								
	Negative	1 (0.5)	144 (79.1)	(80–100)	(87–96)	(50–82)	(96–100)								
All sera (Panbio SI) (N = 182)		24 (13.1)													
	Positive	17 (9.3)	8 (4.4)					70.8	94.9	68.0	95.5				
	Negative	7 (3.8)	150 (82.4)					(49–87)	(90–98)	(47–85)	(91–98)				
Admission sera (Panbio SI) (N = 166)		12 (7.2)													
	Positive	8 (4.8)	7 (4.2)					66.7	95.5	53.3	97.4				
	Negative	4 (2.4)	147 (88.6)					(35–90)	(91–98)	(27–79)	(93–99)				
Convalescent sera (Panbio SI) (N = 129)		20 (15.5)													
	Positive	15 (11.6)	4 (3.1)					75.0	96.3	78.9	95.5				
	Negative	5 (3.9)	105 (81.4)					(51–91)	(91–99)	(54–94)	(90–99)				
Combined CSF (Panbio SI) and sera (Panbio SI)															
CSF and all sera (N = 182)		30 (16.4)													
	Positive	23 (12.6)	5 (2.7)									76.7	96.7	82.1	95.5
	Negative	7 (3.8)	147 (80.8)									(58–90)	(93–99)	(63–94)	(91–98)
CSF and admission sera (N = 166)		23 (13.8)													
	Positive	16 (9.6)	4 (2.4)									69.6	97.2	80.0	95.2
	Negative	7 (4.2)	139 (83.7)									(47–87)	(93–99)	(56–94)	(90–98)
CSF and convalescent sera (N = 129)		23 (17.9)													
	Positive	17 (13.2)	4 (3.1)									73.9	96.2	81.0	94.4
	Negative	6 (4.7)	102 (79.1)									(52–90)	(91–99)	(58–95)	(88–98)
Combined CSF (Panbio RC) and sera (Panbio SI)															
CSF and all sera (N = 182)		30 (16.4)													
	Positive	25 (13.7)	15 (8.2)									83.3	90.1	62.5	96.5
	Negative	5 (2.7)	137 (75.3)									(65–94)	(84–94)	(46–77)	(92–99)
CSF and admission sera (N = 166)		23 (13.9)													
	Positive	21 (12.7)	12 (7.2)									91.3	91.6	63.6	98.5
	Negative	2 (1.2)	131 (78.9)									(72–99)	(86–96)	(45–80)	(95–100)
CSF and convalescent sera (N = 129)		23 (17.8)													
	Positive	19 (14.7)	11 (8.5)									82.6	89.6	63.3	96.0
	Negative	4 (3.1)	95 (73.6)									(61–95)	(82–95)	(44–80)	(90–99)

Diagnostic accuracy compared with the AFRIMS JEV ELISA.

(Continued)

Supplemental Table
Continued

Test results	AFRIMS JEV ELISA		Diagnostic accuracy compared with the AFRIMS JEV ELISA											
			CSF samples				Serum samples				CSF and Serum combined			
	Positive (%)	Negative (%)	% Sens	% Spec	%PPV	%NPV	% Sens	% Spec	%PPV	%NPV	% Sens	% Spec	%PPV	%NPV
Combined CSF (Panbio SI) and sera (4.97 PanBio Units)														
CSF and all sera (N = 182)	29 (15.9)													
Positive	25 (13.7)	38 (20.9)									86.2 (68–96)	75.2 (68–82)	39.7 (28–53)	96.6 (92–99)
Negative	4 (2.2)	115 (63.2)												
CSF and admission sera (N = 166)	23 (13.9)													
Positive	19 (11.4)	29 (17.5)									82.6 (61–95)	79.7 (72–86)	39.6 (26–55)	96.6 (92–99)
Negative	4 (2.4)	114 (68.7)												
CSF and convalescent sera (N = 129)	22 (17.1)													
Positive	19 (14.7)	19 (14.7)									86.4 (65–97)	82.2 (74–89)	50.0 (33–67)	96.7 (91–99)
Negative	3 (2.3)	88 (68.2)												
Combined CSF (Panbio RC) and sera (4.97 PanBio Units)														
CSF and all sera (N = 182)	29 (15.9)													
Positive	25 (13.7)	32 (17.6)									86.2 (68–96)	79.1 (72–85)	43.9 (31–58)	96.8 (92–99)
Negative	4 (2.2)	121 (66.4)												
CSF and admission sera (N = 166)	23 (13.9)													
Positive	21 (12.7)	33 (19.9)									91.3 (72–99)	76.9 (69–84)	38.9 (26–53)	98.2 (94–100)
Negative	2 (1.2)	110 (66.3)												
CSF and convalescent sera (N = 129)	22 (17.1)													
Positive	19 (14.7)	25 (19.4)									86.4 (65–97)	76.6 (68–84)	43.2 (28–59)	96.5 (90–99)
Negative	3 (2.3)	82 (63.6)												

* 95% confidence intervals (95% CI) are indicated in brackets. "Panbio SI" refers to the Panbio standard interpretation, "Panbio RI" to the Panbio interpretation using Ravi criteria and "4.97 PanBio Units" as a ROCC derived optimum cut-off value (see Table 3).
